# Changes in Reaction Time, Balance and Neuroplasticity after Exercise with a Face Mask in Male Adults with Mild COVID-19 Symptoms

**DOI:** 10.3390/healthcare11202800

**Published:** 2023-10-22

**Authors:** Kamil Michalik, Marcin Smolarek, Jacek Borkowski, Miłosz Tchorowski, Natalia Korczuk, Piotr Gorczyca, Natalia Wojtarowicz, Marek Zatoń

**Affiliations:** 1 Department of Human Motor Skills, Faculty of Physical Education and Sport, Wroclaw University of Health and Sport Sciences, 51-612 Wrocław, Poland; marcin.smolarek@awf.wroc.pl; 2 Department of Physiology and Biochemistry, Faculty of Physical Education and Sport, Wroclaw University of Health and Sport Sciences, 51-612 Wrocław, Poland; jacek.borkowski@awf.wroc.pl (J.B.); marek.zaton@awf.wroc.pl (M.Z.); 3 Students Scientific Association Exercise Physiology, Faculty of Physical Education and Sport, Wroclaw University of Health and Sport Sciences, 51-612 Wrocław, Poland; 53875@student.awf.wroc.pl (M.T.); 50771@student.awf.wroc.pl (N.K.); 50883@student.awf.wroc.pl (P.G.); 49171@student.awf.wroc.pl (N.W.)

**Keywords:** COVID-19, surgical face mask, posturography, neuroprotective, BDNF, cortisol

## Abstract

This study compared physiological, perceptual and neuroprotective hormone and metabolite responses and changes in coordination as an effect of aerobic exercise with and without a face mask in people with mild symptoms of COVID-19. Forty men took part in this study. Half declared mild symptoms of SARS-CoV-2 infection in the 6 months before the study (Declared) and the other half did not (Non-declared). In a random order, with a 7-day interval, they performed a 30-min walk on a treadmill at a speed of 6 km/h wearing a surgical face mask (Masked) and without it (Unmasked). The heart rate, heart rate variability, oxygen saturation, lactate concentration and rate of perceived exertion were recorded. The reaction time and balance were measured before and after the exercise. The concentrations of brain-derived neurotrophic factor, testosterone, cortisol, epinephrine and antibodies in the blood serum were determined. Physiological and perceptual responses, reaction times, and balance did not differ between the tested conditions. Three-way RM-ANOVA with post hoc Bonferroni analysis revealed lower post-exercise cortisol concentrations in the Masked and Unmasked conditions in both groups (*p* ≤ 0.001). Asymptomatic infection with this virus is prevalent, and mild COVID-19 causes similar responses to aerobic exercise with a surgical face mask and does not lead to impaired coordination.

## 1. Introduction

At the beginning of 2020, the COVID-19 pandemic caused by the SARS-CoV-2 coronavirus caused a global state of emergency [1]. According to the World Health Organization (WHO) report of 11 September 2023, after more than three years, the COVID-19 pandemic has significantly impacted global public health, with over 770 million confirmed cases of infection and nearly seven million deaths [2]. The disease manifests itself with a range of symptoms, from mild (fever, pain, dizziness, dry cough or fatigue) to severe ones (such as pneumonia or acute respiratory failure with cardiac consequences) [3]. Symptoms of the infection also include disturbances of smell and taste, balance disorders and neurological problems [4,5]. Health researchers found that, while most of the infected population was asymptomatic or had mild symptoms with quick recovery, a portion of the infected population developed long-term symptoms [6]. Lopez-Leon et al. and Costa et al. [7] estimated that approximately 80% of infected patients developed at least one long-term symptom, manifesting mainly in the cardiovascular and respiratory systems, internal organs, central and peripheral nervous systems, and vision. This condition has been called “long COVID-19” or “post-COVID-19 syndrome” by doctors and scientists [8], and the consequences of SARS-CoV-2 infection seem to concern both hospitalized and non-hospitalized patients [9]. Many patients were diagnosed with an extended spectrum of physical, psychiatric, neurological and neuropsychological dysfunctions during the course of the disease and/or 3 to 6 months after recovery [10,11]. Neurological symptoms, such as cognitive impairment during COVID-19 infection and “post-COVID-19 syndrome”, may be due to hypoxia occurring during infection, SARS-CoV-2-mediated neuroinflammation, or the presence of SARS-CoV-2 in brain tissue [12].

One of the common symbols of the pandemic was the introduction of preventive measures by governments, such as isolation, mandatory wearing of protective masks and distancing recommendations, to reduce the number of infections [13]. Also, treatment, vaccination (e.g., mRNA-based) and combinations of vaccinations and non-pharmaceutical interventions should be mentioned [14,15]. Brüssow and Zuber [14] reported in their review that the combination of vaccination and wearing a mask is potentially synergistic, since vaccination protects against disease development to a certain extent, but immunity from infection wanes over a few months after vaccination. The impact of isolation manifested itself, among others, in a change in the habits of the general population, which has negatively impacted health and well-being. As a result, there has been a significant increase in psychological, behavioral and social problems [16] and a decrease in the level of physical activity [17]. These, in turn, are correlated with a higher risk of cardiovascular diseases, shorter life expectancy and lower levels of strength and physical capacity [18]. However, face masks were introduced to limit the excretion of respiratory droplets and thus reduce respiratory viral infections [19]. Surgical face masks are the most commonly used type of face mask, and they limit facial contact with large droplets [20]. Research so far has largely focused on physiological responses (heart rate (HR), heart rate variability (HRV), systolic and diastolic blood pressure, blood oxygen saturation (SaO_2_) and perceptual responses (rating of perceived exertion (RPE)) during incremental and strength exercise in healthy and sick people [13,21,22,23,24]. While some studies have shown that wearing a surgical face mask will have a negative impact on performance and physiological variables [22,25,26], others have shown contrasting results [21,27,28]. These discrepant results between the studies may be attributable to significant methodological differences. Although face masks can effectively reduce virus transmission, wearing them is perceived as uncomfortable [22]. Available data suggest that the adverse effects of using cloth surgical masks during physical activity in healthy individuals are negligible and unlikely to impact exercise tolerance significantly. However, in people with pre-existing cardiopulmonary disease, introducing additional breathing resistance and causing an increased amount of inhaled carbon dioxide, which may be visible in blood gas measurements (increased CO_2_ pressure) may increase the feeling of shortness of breath and therefore affect the ability to exercise [23,29]. Therefore, it is necessary to determine whether using a surgical face mask in people after COVID-19 causes more significant changes in acute and prolonged responses during physical activity, e.g., low-intensity aerobic exercise. One such activity is undoubtedly walking, one of the daily activities recommended for maintaining physical and mental health [30].

Muscle fatigue is an inevitable consequence of physical, occupational or recreational loads [31]. This condition leads to a change in the quality of somatosensory information and its integration within the central nervous system [32] and reduces the potential for generating force by muscles [33]. Altered somatosensory input and decreased neuromuscular control associated with muscle fatigue may increase the likelihood of adverse events, e.g., impaired motor coordination resulting in falls [34]. Moreover, fatigue also leads to longer reaction times [35]. Balance plays a crucial role in human life in physical activities performed as part of everyday duties [36]. The central nervous system’s coordination of vestibular, visual and proprioceptive information is necessary for proper functioning [37]. This fact suggests that COVID-19 may intensify the decline in physical performance, the development of fatigue and the weakening of motor functions [38,39]. People who have experienced olfactory dysfunction or respiratory failure during COVID-19 show balance deficits after recovery, which may indicate less effective peripheral control [40]. Interestingly, it has been found that even mild COVID-19 infection can cause balance disorders in young adults [41]. Furthermore, an association between slowed reaction time and physical and cognitive symptoms after COVID-19 has been reported, suggesting that impaired attention and reaction time are caused by a complex interaction between physical and cognitive symptoms [42,43]. Hence, there is a need to diagnose the long-term effects of this disease and the neurological consequences of COVID-19 on human motor coordination.

It is well known that regular aerobic exercise benefits the body’s functioning and maintains health [44]. Positive adaptations resulting from regular exercise are observed, among others, at the molecular level [45]. This leads to increased physical performance and fitness, improved energy processes, and improved motor coordination, which are associated with more effective central nervous system operation [46]. It has been shown, among other things, that aerobic exercise improves balance [47] and reaction time [46]. Among the mechanisms in which aerobic exercise exerts the so-called neuroplastic effect is brain-derived neurotrophic factor (BDNF) [48]. It has been shown that exercise-induced elevated lactate levels (a result of anaerobic metabolism of muscle glycogen or glucose) also exert neuroplastic effects [49]. In turn, epinephrine affects metabolism and increases lactate concentration during exercise [50]. Moreover, increased testosterone concentration has a positive effect on the level of BDNF and, consequently, on neuroplastic mechanisms [51]. Contrary to the too-high concentration of cortisol (a major glucocorticoid hormone in humans) [52]. Testosterone has been shown to increase neurogenesis in the brains of adult mammals. The neuroprotective effect of this hormone is probably related to the activity of BDNF, but elevated glucocorticoid levels impede neurogenesis [51]. Higher levels of saliva-free cortisol can be reduced via 15 min aerobic exercise, as reported by Ida et al. [53] among depressed patients, and this decrease was accompanied by an improvement in subjective depressive symptoms. We were interested in testosterone concentration because it has been shown that infection with the SARS-CoV-2 virus affects the production of this hormone. It has also been shown that testosterone levels influence the course of COVID-19 disease [54,55]. Toscano-Guerra et al. [55] showed a significant role of testosterone status in the immune responses to COVID-19. Salonia et al. [56] reported interesting results that more than 50% of men who recovered from COVID-19 still showed low circulating testosterone levels. This suggests that males should be regularly tested, because low concentrations of testosterone may negatively impact BDNF levels and neuroplasticity. Finally, examining testosterone levels is important because previous clinical evidence suggests that men with lower testosterone levels at baseline could have worse outcomes because of COVID-19, despite equal comorbidities at presentation [57]. Therefore, the consequences of the COVID-19 pandemic presented above, resulting from a reduction in the level of physical activity and wearing face masks during various forms of physical activity, may lead to a greater level of fatigue and then to a decline in the efficiency of cognitive and motor functions, causing reluctance to exercise. Therefore, it may limit physical activity and/or cause later neurodegenerative problems.

Taking into account the above, this study aimed to compare: (1) current and prolonged physiological, biochemical and perceptual reactions; (2) changes in motor coordination (reaction time and balance); and (3) changes in the concentration of neuroprotective hormones and metabolites (BDNF, lactate, epinephrine, testosterone and cortisol) after 30 min of low-intensity exercise (walking) on a treadmill on a mechanical treadmill with and without a surgical face mask in people who showed symptoms of COVID-19 disease in the last six months. We hypothesize that in people with oligosymptomatic COVID-19, exercise with a surgical face mask will cause increased body load (physiological, biochemical, hormonal and perceptual reactions) during aerobic exercise, resulting in a prolonged reaction time and weakened balance in a standing position. 

## 2. Materials and Methods

### 2.1. Participants

Forty adult men, aged 20–40 years took part in the research. Among them, 20 participants were recruited who declared symptoms of SARS-CoV-2 infection in the 6 months before the study (Declared) and 20 people who did not show symptoms of SARS-CoV-2 infection during that time (Non-declared). Inclusion and exclusion criteria were based on the guidelines presented in the COVID-19 Treatment Guidelines—Clinical Spectrum [58]. Inclusion criteria for the study were as follows: (1) asymptomatic or pre-symptomatic infections: people whose virological test results (i.e., nucleic acid amplification test (NAAT)) were positive for SARS-CoV-2 but did not have symptoms consistent with COVID-19; (2) only mild disease: people who had any signs and symptoms of COVID-19 (e.g., fever, cough, sore throat, malaise, headache, muscle pain, nausea, vomiting, diarrhea, loss of taste and smell), but not shortness of breath or abnormal chest imaging. However, the exclusion criteria from the study included: (1) previous circulatory and respiratory diseases; (2) critical illness: people with respiratory failure, septic shock and/or multi-organ dysfunction after SARS-CoV-2 infection; (3) a need for hospitalization due to COVID-19; (4) people practicing sports professionally; and (5) people who smoke tobacco and use other stimulants. The mean time after infection was equal to 4 ± 1 months. After familiarizing themselves with the procedure, each participant signed a written and informed consent form to participate. The study was approved by the local Research Ethics Committee (17/2022) and was conducted following the Declaration of Helsinki (PN-EN ISO 9001: 2001 certificate). Everyone completed the experiment. Detailed characteristics of the subjects are presented in Table 1.

Prior to the experiment, the G*Power software (version 3.1.9.2; Kiel University, Kiel, Germany) [59] was used to estimate the required sample size for ANOVA with repeated measurements setting a minimum expected effect size (Cohen’s *f*) of 0.5, an α level of 0.05, and a power (1-β) of 0.9. The procedure returned a minimum number of 16 participants per group. 

### 2.2. Study Design

**Figure 1:**
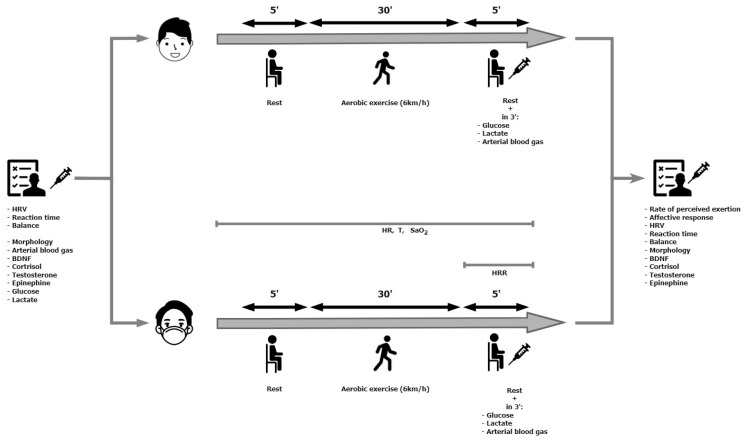
Experimental scheme.

The research was conducted in April 2023 and included three visits to the laboratory. During the first one (the familiarization session), anthropometric, blood pressure and morphology measurements were performed. Also, the participants were allowed to familiarize themselves with the place where the experiment was carried out and the test procedures (measuring reaction time and balance). During the second and third visits (Figure 1), appropriate tests were performed in a randomly selected order using Research Randomizer v4. [60]: walking on a treadmill at a speed of 6 km/h: (1) with a surgical face mask (Masked); (2) without a mask (Unmasked). During exercise, heart rate and blood oxygen saturation were recorded. Reaction time and balance in a standing position were also measured before and after exercise. The heart rate variability in the supine position, blood gases and the concentrations of glucose, lactate, brain-derived eutrophic factor (BDNF), testosterone, cortisol and epinephrine were measured. After completing the exercise, the heart rate recovery, the rate of perceived exertion and the affective response were determined. The subjects were asked not to undertake heavy exercise or to completely give up training in the 24 h before the test. Both tests were performed at the same time of day (08:00–11:00) in laboratory conditions to avoid circadian fluctuations, and the interval was 7 days. The participants were also asked to avoid consuming caffeine and alcohol for 24 h before each experimental session. The subjects came to the laboratory after an overnight fast (11 ± 1 h). To avoid dietary effects on metabolism during exercise, subjects maintained the same diet during the three days preceding each test [61]. The staff were vaccinated and used face masks during all experiments. 

### 2.3. Body Composition Analysis

Height (cm) and body weight (kg) measurements were taken before exercise tests on a WPT 200 medical scale (Radwag, Radom, Poland). Then, the subjects were analysed for body composition using the InBody^®^ 230 analyser (InBody Co., Ltd., Seoul, Republic of Korea). A bioelectrical impedance analysis assessed body composition using two different frequencies (20 kHz and 100 kHz) of five body segments (right and left arm, trunk, right and left leg) using an 8-point touch electrode system. The results were processed using Lookin’Body 120 software (v. MDB_IB120) (InBody Co., Ltd., Seoul, Republic of Korea). Age, height and sex were entered into the analyzer prior to measurement, and fat mass, segmental fat mass, percent body fat (%FM), skeletal muscle mass, segmental fat-free mass, and total body water were assessed. 

### 2.4. Measurement of Choice Reaction Time 

The choice reaction time (CRT) was measured using MCZR/ATB 1.0 m (ATB Info-EleKtro, Zabrze, Poland) and the procedure described by Hebisz et al. [62]. Measurements were taken at rest before the exercise and 10 min after its completion. The reaction time meter consisted of a central control panel connected to the visual and auditory stimuli navigation signals and a stimulus reception panel. The display emitted visual stimuli in three colors: red, orange and green. The stimulus reception panel consisted of two manual buttons (one on the right and one on the left) placed on cables with a jack plug. The participant sat on a chair 3 m from the display during the measurement. Participants were asked to respond as quickly as possible by pressing a button with their right hand when a red light appeared and with their left hand when a green light appeared. Intentionally, the button in the right hand was red, while the one in the left hand was green. Participants were not expected to respond to the orange light, and a response was an error. Each participant underwent a familiarization test measurement that lasted 40 s, which consisted of 10 stimuli and was performed only once. However, the main measurement lasted 120 s and included 27 stimuli arranged in a specific order and occurring at a specific time. Seventeen stimuli were to be responded to, and 10 were not. The measurement assessed the mean CRT (time calculated from all reactions), minimum RT (shortest reaction), maximum RT (longest reaction), and number of correct reactions. 

### 2.5. Balance Assessment 

Balance was assessed before and 15 min after the completion of exercise. The subject stood barefoot on the ALFA Stabilometric Platform with dimensions of 550 × 550 × 80 mm (AC International East, Knurów, Poland) with strain gauge sensors placed in the corners recording the central pressure of the feet on the ground of the general center of pressure (OSC projection), as well as its displacement in the sagittal X axis (i.e., left-right) and frontal Y (i.e., front-back). The platform was placed 150 cm from the wall, and the tests were carried out in an empty, quiet room. The test was performed with eyes open (EO) and then with eyes closed (EC). Each examination lasted 30 s. The evaluation included the length of the path drawn in front of the projection of the center of gravity (COP_L_) and the surface area on the coordinate axis (COP_A_).

### 2.6. Biochemical Measurements 

In order to determine the concentration of brain-derived neurotrophic factor (BDNF), testosterone (T), cortisol (C) and epinephrine (E) in blood serum, blood was collected from an antecubital vein at rest before and 30 min after its completion. The material for determination was collected in Sarstedt tubes with serum granules at a volume of 10 mL (Stamar, Dąbrowa Górnicza, Poland). After clot formation, the samples were centrifuged for 10 min at 3000 rpm (Eppendorf Centrifuge 5810, Hamburg, Germany) at 4 °C. Separated serum samples were frozen and kept at −80 °C for later analysis. Serum samples were divided into aliquots to avoid repeated thawing. Following the manufacturer’s instructions, BDNF concentration was determined using the Sandwich ELISA test (EIAAB Science Inc., Wuhan, China), catalogue number E0011H. Testosterone, cortisol and epinephrine concentrations were determined using the competitive ELISA method using kits (EIAAB Science Inc, Wuhan, China) catalogue numbers E0458GE for testosterone, E0462GE for cortisol, and E0858GE for epinephrine. Moreover, the level of IgG antibodies against the nucleocapsid antigen of the SARS-CoV-2 virus was measured by means of ELISA (Wuhan Fine Biotech Co., Ltd., Wuhan, China) catalogue number EH4397. All measurements were performed in duplicate. The measurements were performed using an Epoch plate reader from BioTek, Charlotte, VT, USA (microplate spectrophotometer) and the Gen5 2.0 program from the same company.

Capillary blood was collected from the fingertip of the hand (before exercise at rest and in the third minute after its completion) in order to determine: (1) white blood cells (WBC), red blood cells (RBC), hemoglobin (Hb) and hematocrit (Ht) concentration using the ABX Micros OT.16 apparatus (Horiba Medical, Kyoto, Japan); (2) acid–base balance using the RapidLab 348 analyzer (Bayer, Leverkusen, Germany), blood pH, which was converted to the concentration of hydrogen ions ([H^+^]) according to the formula: [H^+^] = 10^−pH^, partial pressure of oxygen (pO_2_) and carbon dioxide (pCO_2_), and bicarbonate concentration [HCO_3_^−^]; (3) glucose (Glu) concentration using the On Call^®^ GK Dual device (ACON Laboratories, San Diego, CA, USA); (4) lactate concentration ([La]) using an LP 400 photometer and using an LP 140 (Dr Lange, Berlin, Germany). 

### 2.7. Prolonged Exercise with and without a Surgical Face Mask

The subjects’ task was to perform a 30-min exercise on a SEGTA7720 mechanical treadmill (InSportLine, Prague, Czech Republic). The exercise was performed at a 6 km/h speed to simulate the walking intensity. The total distance covered in a single session was 3 km. One of the two sessions was performed with a disposable surgical face mask (APC System Med., Nowy Sącz, Poland). It was a non-woven, three-layer mask, certified (PN-EN-14683:2019+AC:2019, type I) with a BFE >95% (bacterial filtration efficiency).

### 2.8. Physiological Measurements

Systolic blood pressure (SBP) and diastolic blood pressure (DBP) were measured at rest before exercise using an anoreid sphygmomanometer (Riester, Jungingen, Germany).

Heart rate (HR) was recorded continuously to the nearest millisecond using a V800 sport tester (Polar Electro Oy, Kempele, Finland. Based on the recordings, the average (HRav) and peak (HRpeak) heart rate, as well as heart rate recovery (HRR), were determined 60 s after the end of the exercise (in a sitting position). The predicted maximal heart rate (HRmax_pred_) was calculated using the formula [63]: 208 − 0.7 ∙ age (years). On this basis, the percentage of heart rate (%HR_PRED_) in relation to HRmax_pred_ was calculated.

Heart rate variability (HRV) was measured in the supine position, and data collected from the last two minutes of a 5-min period were analyzed. HRV was analyzed using specially designed software (Kubios HRV Analysis version 2.2, The Biomedical Signals Analysis Group, University of Kuopio, Kuopio, Finland), and the time and frequency parameters of sinus heart rate variability were calculated. The following were analyzed: the square root of the average sum of squares of differences between successive intervals (rMSSD) and the fast Fourier transform spectrum parameter, namely the power ratio of low- and high-frequency spectra (LF/HF). 

Blood oxygen saturation (SaO_2_av) was continuously recorded non-invasively during exercise via optical transmittance based on digital technology using a CMS50D pulse oximeter (Contec, Qinhuangdao, China) attached to the right hand’s middle finger.

### 2.9. Psychological Responses

The Borg scale was used to assess subjective feelings of exertion (RPE). The scale comprises 15 levels (6–20) [64]. The subjects rated their perceived effort immediately after completing the exercise.

Affective responses (AR) were defined using the feeling scale as a feeling of pleasure or dissatisfaction (−5/+5) [65]. An 11-item bipolar scale with a dimensional model ranging from +5 to −5, multiple choice, is commonly used to measure affective value during exercise.

### 2.10. Statistical Analysis 

IBM SPSS Statistics version 26 software package (IBM, Inc., Chicago, IL, USA) was used to analyze the data statistically. The results are presented as the arithmetic mean ± standard deviation (Mean± SD) and 95% confidence interval (95% CI). The Shapiro–Wilk test was used to assess the normality of the distribution of the examined features, and the homogeneity of variance was assessed using the Levene test. Student’s *t*-test for independent samples assessed differences in parameters characterising the groups. A three-way RM-ANOVA with *Time* (pre-post), *Group* (declared, non-declared CV-19) and *Condition* (Masked, Unmasked) was conducted for metabolic (Glu, [La^−^], [H^+^], [HCO_3_^−^]), hormonal (BDNF, T, C and E), and physiological responses (rMSSD, LF/HF). However, a two-way ANOVA with *Group* (declared, non-declared CV-19) and *Conditions* (Masked, Unmasked) was conducted for physiological (HRav, %HR_PRED,_ HRpeak, HRR and SaO_2_av) and psychological responses (RPE, affective responses). When a significant F ratio value was obtained, a post hoc Bonferroni test was performed. The effect size was estimated using partial eta square (η^2^), classified as small (0.2 < η^2^ < 0.49), medium (0.5 < η^2^ < 0.79), or large (η^2^ ≤ 0.8) [66]. A *p* < 0.05 was considered statistically significant.

## 3. Results

The groups differed statistically in terms of the percentage of adipose tissue (*p* < 0.05, t = 2.02) and diastolic blood pressure (*p* < 0.05, t = 2.04). All the subjects had IgG antibodies against the nucleocapsid antigen of SARS-CoV-2, the average concentration of which was 4.1 ± 1.1 (95% CI 3.8–4.3) (µg/mL). 

Two-way analysis of variance revealed that there were no significant differences between other physiological (average and peak heart rate, percentage of predicted maximal heart rate, heart rate recovery, average oxygen saturation) and psychological (rating of perceived impression, affective responses) responses to the 30-min walk (Table 2). Moreover, the three-way RM-ANOVA showed that the parameters characterising HRV (rMSSD and LF/HF) did not differ statistically significantly (Table 2).

Taking into account the reaction time, the analysis showed a statistically significant main effect of the Time x Condition interaction (F_(1,76)_ = 4.0, *p* ≤ 0.05, η^2^ = 0.05) for RTmax and a main effect of Time (F_(1,76)_ = 18.87, *p* ≤ 0.001, η^2^ = 0.20) for NCR (Table 3). However, post hoc analysis did not reveal intra- or inter-group differences.

Table 4 shows the path length and surface area of the COP with the eyes open and closed. The analysis revealed statistically significant main effects of Time for EC COP_L_ (F_(1,76)_ = 10.28, *p* ≤ 0.01, η^2^ = 0.12) and EO COPA (F_(1,76)_ = 11.33, *p* ≤ 0.01, η^2^ = 0.09). Also, in the case of these parameters, post hoc analysis did not reveal intra- and inter-group differences. No statistically significant differences were identified between EO COP_L_ and EC COP_A_.

Table 5 shows the metabolic and hormonal parameters measured before and after exercise. A statistically significant main effect of Time occurred for Glu (F_(1,76)_ = 15.31, *p* ≤ 0.001, η^2^ = 0.17), [La^-^] (F_(1,76)_ = 4.78, *p* ≤ 0.05, η^2^ = 0.06), [H^+^] (F_(1,76)_ = 155.18, *p* ≤ 0.001, η^2^ = 0.67) and [HCO_3_^-^] (F_(1,76)_ = 14.18, *p* ≤ 0.001, η^2^ = 0.16). Only for the [H^+^] concentration, the Bonferroni post hoc test showed a statistically significant reduction after exercise for both groups and test conditions (*p* ≤ 0.001). Moreover, for [La^-^], the three-way RM-ANOVA showed a statistically significant Time x Group interaction (F_(1,76)_ = 5.08, *p* ≤ 0.05, η^2^ = 0.06). We found a statistically significant main effect of Time for cortisol (F_(1,76)_ = 145.3, *p* ≤ 0.001, η^2^ = 0.66) and testosterone (F_(1,76)_ = 4.51, *p* ≤ 0.05, η^2^ = 0.06). Furthermore, post hoc analysis significantly reduced the post-exercise cortisol concentration for all four experimental conditions (*p* ≤ 0.001). At the same time, no statistically significant differences in BDNF and epinephrine concentrations were found. 

## 4. Discussion

This study aimed to determine whether, in people who have had COVID-19 in the last 6 months, wearing a surgical face mask while walking at a speed of 6 km/h leads to increased body load, impaired balance and reaction time, and how it affects the concentration of neuroprotective hormones and metabolites. An unexpected result was that all participants recruited for this experiment, regardless of whether they reported symptoms of the disease in the questionnaire, had IgG antibodies against the nucleocapsid antigen of the SARS-CoV-2 virus and therefore had contact with the virus regardless of the vaccine preparation received. However, we do not know the variant of the virus causing COVID-19 symptoms in the subjects. At the same time, we did not demonstrate changes in physiological parameters (HR, HRR, HRV and SaO_2_av), metabolic parameters ([La^-^], [H^+^] and Glu) and perception parameters (RPE, AR), or deterioration of reaction time and balance. Using a surgical face mask in the group declaring mild symptoms of COVID-19 did not affect the post-exercise concentration of BDNF, adrenaline and testosterone. However, exercise had a positive effect on reducing cortisol levels in all test conditions in both groups. 

Contrary to our hypothesis, wearing a surgical face mask during low-intensity exercise in the Declared group did not appear to have a negative effect. This hypothesis was based on a previous comment [29] suggesting that exercising with a face mask may pose health risks and stress various physiological systems, such as the respiratory, circulatory and immune systems, due to hypercapnia (i.e., increase in carbon dioxide concentration in the blood). First, in the present study, we did not observe increased pCO_2_ in the blood and respiratory acidosis because the concentration of [H^+^] ions after exercise decreased in all test conditions and was within the physiological norm. Our results are consistent with those presented by Ogawa et al. [24], where no higher level of end tidal partial pressure carbon dioxide (P_ET_CO_2_) was found during the progressive test with a face mask. Therefore, it is not a factor provoking additional load on the body’s activities during low-intensity exercise (<55% HRmax, RPE 8–9). Second, we showed no differences in blood oxygen saturation and heart rate in both groups, which confirms previous studies that compared the effects of wearing cloth masks [21] or surgical masks [21,28] during a progressive test on a cycle ergometer in healthy people. Third, our HRV analysis confirmed a similar autonomic response in people after mild COVID-19, regardless of whether they were masked. Our reported results are consistent with previous suggestions by Ramos-Campo et al. [13] that the type of face mask (surgical, FFP2) does not influence changes in HRV after resistance exercise in people with sarcopenia. The above statements are also confirmed by the lack of significant differences in the restorative heart rate regulated by the autonomic nervous system [67]. Fourth and finally, the perceptual responses in our studies did not differ significantly. This may be related to the suggestion of Poon et al. [68] that physical work intensity ≥75% VO_2_max (maximal oxygen uptake) results in increased perceived exertion. Other studies have observed an increased sense of perceived exertion and symptoms of shortness of breath during exercise with progressively increased intensity [22]. This clearly indicates that people can wear a surgical face mask during low-intensity exercise, such as walking at a speed of 6 km/h, regardless of their history of mild COVID-19. However, subsequent studies should consider other types of face masks, e.g., N95 or FFF2, other types of exercise and the severity of COVID-19 disease in the examined people. 

As previous studies have shown [10,69,70], post-COVID-19 syndrome is characterized by multiple symptoms such as fatigue, dizziness, problems with concentration, memory and executive functions that affect all daily activities. In our research, we used reaction time measurement because it is associated with a decrease in the speed of information processing [71]. It has also been shown that reaction time measurement can be used to estimate the risk of death from COVID-19 [71]. Psychologists pay attention to reaction time as a tool for assessing the limits of the functioning of the nervous system in relation to the complex reasoning process [72]. Initially, we assumed that people with a mild course of COVID-19 would experience greater fatigue changes, which would manifest themselves in an extended reaction time after exercise. Our reported reaction times at rest and after exercise are similar to those previously published by Hebisz et al. [62] in athletes, i.e., mountain bikers. This includes average, minimum and maximum response times. Moreover, our results confirm the conclusion of Santoyo-Mora et al. [42], who stated that the degree of infection is important because people with mild and moderate COVID-19 show similar effectiveness in decision-making and information processing speed to people who have never been infected. In their research, the speed of information processing, measured by a simple reaction time test, revealed that in patients who had experienced a severe and critical condition related to COVID-19, the reaction time was longer compared to people who had never been infected (Z = −3.652, *p* < 0.001) and cases after COVID-19 of mild and moderate severity (Z = −2.767, *p* = 0.006) [42]. Maiorana et al. [43] also studied reaction time, but their study was based on a web-based Visual Detection Task (VDT). They found that physical and cognitive symptom factors predicted reaction times in the VDT. In particular, by analysing physical and cognitive factors, they proved that learning disorders (cognitive factor), visual disturbances and headaches (physical factor) were the most important predictors of slower reaction time [43]. However, it should be taken into account that, in the case of our study, the full clinical analysis of the participants was not assessed because their clinical history included in the study was based only on an oral interview. Although Santoyo-Mora et al.’s [42] study was also based on an oral interview, in our research, we additionally performed an IgG antibody test, which indicated that all subjects had contact with the virus, regardless of the vaccine preparation. This may suggest that subsequent studies should be based on extended diagnostics of potential participants, also taking into account the recovery period of the recruited participants. As shown in the studies mentioned above by Santoyo-Mora et al. [40], for mild and moderate cases, it was, on average, 8.70 ± 5.04 months and 11.25 ± 5.29 months for severe cases.

Another examined aspect was maintaining balance. Maintaining balance is a complex process that requires the integration of various stimuli to function properly. Balance also involves stability, i.e., the ability to maintain and regain the original state during or after a specific movement/exertion. The efficiency of the postural control system enables the proper maintenance of static balance, i.e., while standing and dynamic, i.e., while moving, despite the influence of external stimuli. This is expressed by assessing the foot’s center of pressure in the foot support area [73]. This seems particularly important in recent years because early detection of neurological deficits in patients who have recovered from COVID-19 may enable the early implementation of therapeutic procedures [40]. Therefore, monitoring for the short- and long-term complications of SARS-CoV-2 infection should be recommended [40]. We speculated that SARS-CoV-2 disease may have impacted post-exercise fatigue and neuromuscular control, leading to balance problems in participants with a history of mild symptoms of COVID-19. However, in our own research, we did not find any balance disorders with eyes open and closed before and after exercise in the Masked condition in this group compared to the Non-declared group. The results of our research are inconsistent with the results presented by Dzięcioł-Anikiej et al. [37]. Their findings indicated that the balance in patients after COVID-19 was disturbed compared to that of people from the control group. Worse results were obtained in patients regarding the length of the center of gravity path and the surface area determined via COP [37]. A study by Guzik et al. [41], which examined 50 patients with mild COVID-19 compared to the control group, showed that people from the experimental group obtained higher results in the assessment of path length and average COP speed when tested with open eyes (*p* < 0.001). However, in assessing balance with eyes closed, the study group obtained significantly higher results in assessing path length (*p* = 0.035) and average speed (*p* = 0.026). Therefore, the presented research does not confirm that patients with mild COVID-19 experience balance disorders detectable in posturography tests. 

Previous studies have mentioned that there is a presumed increased risk of developing neurodegenerative diseases, including Alzheimer’s disease, Parkinson’s disease and multiple sclerosis, following SARS-CoV-2 infection [40,74,75,76]. Therefore, actions should be taken to neutralize the negative effects of the pandemic if they occur in people infected with mild symptoms. For this purpose, we checked whether low-intensity exercise with a face mask affects the level of selected metabolites and hormones with neuroplastic effects, such as BDNF, which is a protein synthesized in the central and peripheral nervous systems in neuron endings. At the same time, it is responsible for the development and growth of neurons, learning and memory processes, apoptosis, neurogenesis and neuroregeneration [77]. Our reported BDNF levels within the physiological range and the previously mentioned lack of deterioration in reaction time and coordination indicate that the mild course of the disease probably did not adversely affect the functioning of the nervous system. Undoubtedly, these studies are critical from a societal point of view, as a linear and inverse relationship between BDNF and adverse outcomes in COVID-19 patients has been reported [78]. The results of these authors also show that the concentration of circulating BDNF decreases with age and increases BMI. Moreover, in the case of COVID-19 disease, a hypothetical model suggested that the downregulation of angiotensin-converting enzyme 2 in the brain by SARS-CoV-2 inhibits the release of neurotrophic factors, such as BDNF [79]. Although we indicate that low-intensity exercise in the Masked condition does not increase the level of BDNF in both tested groups and, therefore, has a neuroplastic effect, future studies should include an experimental model based on higher-intensity exercise sessions that can promote higher BDNF concentrations [80]. 

As for blood lactate and glucose concentrations, our results suggest that wearing a face mask during a low-intensity aerobic session causes similar metabolic stress, as evidenced by the similar values of these markers in both groups. Also, similar results were obtained by Ramos-Campo et al. [13], who reported no differences in lactate concentration in a resistance session performed with a face mask in patients with sarcopenia. However, we did not demonstrate any differences in the concentrations of adrenaline and testosterone, which are within the normal range. It seems that low-intensity exercise (<55% HRmax, RPE 8–9) was crucial here, and wearing a surgical face mask did not cause additional stress that would result in an increased sympathetic and anabolic response of the body. However, it is worth noting that we observed a significant reduction in cortisol concentration after exercise in all test conditions. This fact is not surprising because only physical activity that exceeds 60% of the maximum oxygen uptake induces the release of cortisol above the resting level [81]. It has been shown that low testosterone levels may be associated with the severity of infection with the SARS-CoV-2 virus [82]. The results of our research are consistent with other recommendations stating that aerobic physical activity performed 4 times a week for 20–40 min reduces cortisol levels and improves sleep quality [83]. People after COVID-19 are especially recommended to be physically active during the day, in sunlight, outdoors, e.g., in the forest [84], and not immediately before bedtime [85].

Despite the interesting results presented in this study, some limitations should be pointed out. We only studied men aged 20–40 years, so the results can be extrapolated cautiously to this population. Subsequent experiments should also include women and people of different ages and somatic profiles. Moreover, we included only patients with mild COVID-19 and tested the most commonly used surgical face masks. The virus variant certainly influenced the intensity of the symptoms, but we simply excluded people who had COVID-19 with severe symptoms. Determining the virus variant several months after infection is almost impossible without the use of highly specific antibodies capable of recognizing the characteristic epitope of the viral protein. Our goal was to confirm infection with this virus. Therefore, we decided to use a test that determines anti-nucleocapsid antibodies, which are a less variable part of the virus. This protein does not appear in the body as an antigen after vaccination with mRNA vaccines. 

## 5. Conclusions

Antibodies against the nucleocapsid antigen of SARS-CoV-2 were found in all subjects, suggesting that asymptomatic infection with this virus is very common. Our research shows that, among people who had mild COVID-19 symptoms in the last six months (Declared), acute and prolonged responses to aerobic exercise with or without a surgical mask are similar to the Non-declared group. This applies to both current and prolonged physiological, biochemical and perceptual reactions. We also state that these people do not have resting or post-exertion disorders of motor coordination measured via reaction time and posturography tests. However, the applied low-intensity exercise does not affect the concentration of markers such as BDNF, lactate, testosterone or epinephrine, which have a direct or indirect neurotrophic effect. However, it positively reduces cortisol levels without the negative impact of wearing a surgical mask. 

## Figures and Tables

**Table 1:** Participants’ characteristics—overall and both groups.

Variable	Overall (n = 40)		Declared (n = 20)		Non-Declared (n = 20)	
	Mean ± SD	95% CI	Mean ± SD	95% CI	Mean ± SD	95% CI
Age (years)	27.6 ± 6.1	26.2–29.0	27.8 ± 5.5	26.0–29.6	27.4 ± 6.8	25.2–29.6
Height (cm)	181.1 ± 5.3	179.9–182.3	182.2 ± 4.1	180.9–183.5	180.0 ± 6.1	178.1–182.0
Weight (kg)	81.4 ± 13.0	78.6–84.3	83.9 ± 14.8	79.2–88.7	78.9 ± 10.4	75.6–82.3
BMI (kg∙m^−2^)	24.8 ± 3.9	24.0–25.7	25.3 ± 4.1	23.9–26.6	24.4 ± 3.7	23.3–25.6
FM (%)	15.3 ± 6.9	13.8–16.9	16.9 ± 6.6 *	14.8–19.0	13.8 ± 6.9	11.6–16.0
HRmax pred (bpm)	189 ± 5	188–190	189 ± 5	187–190	189 ± 5	187–190
SBP (mmHg)	117.2 ± 15.0	113.9–120.5	117.6 ± 15.5	112.7–122.5	116.8 ± 14.6	112.1–121.5
DBP (mmHg)	69.0 ± 9.0	67.0–71.0	71.0 ± 9.3 *	68.0–73.9	67.0 ± 8.4	64.3–69.6
Hb (g∙dL^−1^)	14.4 ± 1.0	14.2–14.7	14.4 ± 0.8	14.1–14.7	14.5 ± 1.1	14.2–14.8
Ht (%)	43.8 ± 3.7	43.0–44.6	43.3 ± 4.4	41.9–44.7	44.3 ± 2.7	43.4–45.1
RBC (10^6^∙mm^−3^)	4.8 ± 0.3	4.8–4.9	4.8 ± 0.3	4.7–4.9	4.9 ± 0.3	4.8–5.0
WBC (10^3^∙mm^−3^)	5.3 ± 1.1	5.1–5.5	5.2 ± 1.1	4.8–5.5	5.4 ± 1.1	5.1–5.8

BMI—body mass index; FM—percentage of fat mass; SBP—systolic blood pressure; DBP—diastolic blood pressure; HRmax pred—predicted maximal heart; Hb—haemoglobin concentration; Ht—hematocrit; RBC—red blood cells; WBC—white blood cells. *—statistical significant difference between groups (*p* ≤ 0.05).

**Table 2:** Acute physiological and psychological responses to aerobic exercise with and without a face mask.

Variable	Declared				Non-Declared			
	Masked		Unmasked		Masked		Unmasked	
	Mean ± SD	95% CI	Mean ± SD	95% CI	Mean ± SD	95% CI	Mean ± SD	95% CI
**Physiological**								
HRav [bpm]	103 ± 9	98–107	99 ± 8	96–103	102 ± 14	96–109	104 ± 14	97–110
%HR_PRED_ [%]	53.9 ± 4.7	51.7–56.2	52.4 ± 4.4	50.4–54.8	54.2 ± 7.5	50.7–57.7	55.0 ± 7.4	51.6–58.5
HRpeak [bpm]	113 ± 11	108–119	110 ± 9	105–114	113 ± 14	107–120	114 ± 14	108–121
HRR [bpm]	81 ± 11	76–86	79 ± 12	73–84	82 ± 18	74–91	81 ± 16	74–89
rMSSD pre [ms]	53.9 ± 42.6	34.0–73.8	71.0 ± 58.2	43.8–98.3	66.7 ± 48.1	44.2–89.2	71.9 ± 38.5	53.9–89.9
rMSSD post [ms]	54.0 ± 27.3	41.2–66.7	66.4 ± 54.6	40.9–92.0	64.5 ± 59.5	36.7–92.4	61.5 ± 39.1	43.2–79.7
LF/HF pre	2.4 ± 2.3	1.3–3.4	2.5 ± 3.5	0.9–4.2	3.1 ± 4.9	0.8–5.4	2.5 ± 4.5	0.4–4.7
LF/HF post	2.5 ± 3.0	1.1–3.9	3.0 ± 4.3	1.0–5.0	3.2 ± 5.3	0.7–5.7	2.6 ± 4.0	0.7–4.5
SaO_2av_ [%]	97.3 ± 0.8	96.9–97.7	97.5 ± 0.9	97.1–97.9	97.1 ± 1.0	96.7–97.5	97.2 ± 0.8	96.8–97.5
**Psychological**								
RPE [6–20]	9.1 ± 1.9	8.2–10.0	8.7 ± 2.2	7.6–9.7	8.9 ± 2.2	7.9–9.9	8.1 ± 2.2	7.0–9.1
AR [−5 to +5]	2.4 ± 2.1	1.4–3.3	3.1 ± 1.9	2.2–4.0	2.5–2.0	1.5–3.4	3.2 ± 1.8	2.3–4.1

HRav—average heart rate; %HRmax—percentage of predicted maximal heart rate; HRpeak—the highest heart rate during exercise; HRR—heart rate recovery; rMSSD—square root of the mean squared difference between successive normal-to-normal RR intervals; LF/HF—low to high frequency ratio; SaO_2_av—average oxygen saturation; RPE—rating of perceived exertion; AR—affective responses.

**Table 3:** Time reaction before and after aerobic exercise with and without a face mask.

Variable	Declared				Non-Declared			
	Masked		Unmasked		Masked		Unmasked	
	Mean ± SD	95%CI	Mean ± SD	95%CI	Mean ± SD	95%CI	Mean ± SD	95%CI
RTav pre [s]	0.34 ± 0.04	0.32–0.36	0.35 ± 0.04	0.33–0.36	0.34 ± 0.03	0.32–0.35	0.34 ± 0.03	0.32–0.35
RTav post [s]	0.34 ± 0.04	0.33–0.36	0.35 ± 0.04	0.33–0.37	0.34 ± 0.03	0.32–0.36	0.34 ± 0.04	0.32–0.36
RTmin pre [s]	0.24 ± 0.03	0.22–0.26	0.25 ± 0.02	0.24–0.26	0.24 ± 0.03	0.22–0.25	0.24 ± 0.03	0.23–0.25
RTmin post [s]	0.25 ± 0.02	0.24–0.26	0.25 ± 0.03	0.23–0.26	0.23 ± 0.02	0.22–0.24	0.24 ± 0.04	0.22–0.26
RTmax pre [s]	0.51 ± 0.10	0.47–0.56	0.53 ± 0.12	0.47–0.58	0.51 ± 0.08	0.47–0.54	0.48 ± 0.08	0.44–0.52
RTmax post [s]	0.49 ± 0.09	0.45–0.53	0.53 ± 0.10	0.48–0.58	0.48 ± 0.08	0.44–0.52	0.53 ± 0.12	0.48–0.59
NCR pre [n]	16.3 ± 0.7	15.9–16.6	16.5 ± 0.8	16.1–16.9	16.2 ± 0.9	15.8–16.6	16.4 ± 1.2	15.8–17.0
NCR post [n]	16.9 ± 0.4	16.7–17.0	16.8 ± 0.4	16.6–17.0	16.7 ± 0.7	16.4–17.0	16.7 ± 0.5	16.5–16.9

RTav—average choice reaction time; RTmin—minimal/shortest choice reaction time; RTmax—maximal/longest choice reaction time; NCR—number of correct reactions.

**Table 4:** Post urography parameters before and after aerobic exercise with and without a face mask.

Variable	Declared				Non-Declared			
	Masked		Unmasked		Masked		Unmasked	
	Mean ± SD	95%CI	Mean ± SD	95%CI	Mean ± SD	95%CI	Mean ± SD	95%CI
EO COP_L_ pre [cm]	22.5 ± 4.9	20.2–24.8	22.3 ± 6.9	19.1–25.6	23.9 ± 9.7	19.4–28.4	20.9 ± 7.0	17.6–24.1
EO COP_L_ post [cm]	23.5 ± 7.5	20.0–27.0	22.9 ± 5.6	20.3–25.6	22.2 ± 5.1	19.8–24.6	20.4 ± 5.3	18.0–22.9
EC COP_L_ pre [cm]	29.0 ± 10.1	24.2–33.7	33.5 ± 18.6	24.8–42.3	31.0 ± 12.5	25.1–36.9	31. ± 15.4	24.2–38.6
EC COP_L_ post [cm]	17.5 ± 10.8	22.4–32.6	28.1 ± 10.0	23.4–32.8	27.8 ± 9.6	23.2–32.3	19.6 ± 14.4	22.8–36.4
EO COP_A_ pre [cm^2^]	2.5 ± 1.8	1.6–3.3	2.0 ± 1.3	1.4–2.6	1.5 ± 0.9	1.1–1.9	1.6 ± 1.0	1.2–2.1
EO COP_A_ post [cm^2^]	2.8 ± 1.8	1.9–3.6	3.0 ± 2.3	1.9–4.1	1.9 ± 0.9	1.5–2.4	2.1 ± 1.5	1.4–2.8
EC COP_A_ pre [cm^2^]	2.1 ± 0.9	1.6–2.5	2.9 ± 2.4	1.8–4.0	1.6 ± 0.7	1.2–1.9	1.9 ± 1.0	1.4–2.3
EC COP_A_ post [cm^2^]	2.5 ± 2.0	1.5–3.4	2.4 ± 1.6	1.7–3.2	1.7 ± 1.1	1.2–2.2	1.8 ± 1.2	1.3–2.4

COP_L_—centre of pressure pathway length; COP_A_—center of pressure surface area; EO—eyes open; EC—eyes closed.

**Table 5:** Acute metabolic and hormonal responses to aerobic exercise with and without a face mask.

Variable	Declared				Non-Declared			
	Masked		Unmasked		Masked		Unmasked	
	Mean ± SD	95%CI	Mean ± SD	95%CI	Mean ± SD	95%CI	Mean ± SD	95%CI
**Metabolic**								
Glu pre [mg∙dL^−1^]	99.7 ± 7.9	96.0–103.3	95.2 ± 5.9	92.4–97.9	97.5 ± 7.8	93.8–101.2	97.0 ± 9.5	92.5–101.5
Glu post [mg∙dL^−1^]	96.4 ± 8.1	92.6–100.1	94.0 ± 8.8	89.8–98.1	93.4 ± 9.1	89.1–97.6	91.3 ± 8.3	87.4–95.1
[La^−^] pre [mmol∙L^−1^]	1.3 ± 0.4	1.1–1.5	1.1 ± 0.5	0.9–1.4	1.0 ± 0.4	0.8–1.2	1.0 ± 0.6	0.8–1.3
[La^−^] post [mmol∙L^−1^]	1.2 ± 0.3	1.0–1.3	0.8 ± 0.5	0.6–1.1	1.0 ± 0.5	0.8–1.2	1.0 ± 0.7	0.8–1.4
[H^+^] pre [nmol∙L^−1^]	41.0 ± 1.9	40.1–41.8	40.4 ± 1.7	39.6–41.1	40.9 ± 1.9	40.0–41.7	40.6 ± 1.1	40.1–41.1
[H^+^] post [nmol∙L^−1^]	38.9 ± 1.3 ^*^	38.2–39.5	38.4 ± 1.6 ^*^	37.6–39.1	39.1 ± 1.4 ^*^	38.4–39.8	39.2 ± 1.4 ^*^	38.6–39.9
pCO_2_ pre [mm Hg]	42.6 ± 3.3	41.0–44.1	42.7 ± 3.3	41.1–44.2	41.4 ± 2.2	40.4–42.5	41.4 ± 2.3	40.4–42.5
pCO_2_ post [mm Hg]	40.9 ± 2.2	39.8–41.9	41.1 ± 2.1	40.1–42.0	40.3 ± 2.1	39.3–41.3	40.4 ± 3.6	38.7–42.1
[HCO_3_^−^] pre [mmol∙L^−1^]	25.1 ± 1.6	24.3–25.8	25.5 ± 1.6	24.8–26.2	24.5 ± 1.3	23.9–25.1	24.6 ± 1.2	24.1–25.1
[HCO_3_^−^] post [mmol∙L^−1^]	25.3 ± 1.1	24.8–25.8	25.8 ± 1.2	25.3–26.4	24.9 ± 1.3	24.3–25.5	25.1 ± 1.0	24.7–25.6
**Hormonal**								
BDNF pre [ng∙mL^−1^]	277.7 ± 105.9	228.1–327.2	256.0 ± 95.4	211.3–300.6	270.9 ± 98.7	224.7–317.0	266.2 ± 116.2	211.8–320.6
BDNF post [ng∙mL^−1^]	270.1 ± 116.9	215.4–324.8	254.5 ± 118.5	199.0–309.9	250.3–120.5	193.9–306.8	256.0 ± 108.1	205.4–306.6
T pre [ng∙dL^−1^]	888.6 ± 278.7	758.1–1019.0	892.9 ± 271.8	765.7–1020.1	1074.2 ± 319.4	924.7–1223.7	1012.5 ± 317.7	863.8–1161.2
T post [ng∙dL^−1^]	855.2 ± 246.5	739.8–970.5	805.9 ± 305.2	663.1–948.8	1048.1 ± 303.2	906.2–1190.0	969.5 ± 367.3	797.5–1141.4
C pre [ng∙mL^−1^]	164.8 ± 62.5	135.6–194	149.8 ± 68.1	117.9–181.7	155.2 ± 74.3	120.4–190.0	145.6 ± 84.7	106.0–185.2
C post [ng∙mL^−1^]	93.0 ± 25.2 *	76.6–109.5	82.4 ± 37.0 *	65.1–99.7	90.1 ± 44.7 *	69.2–111.1	87.2 ± 42.6 *	67.2–107.1
E pre [pg∙mL^−1^]	64.2 ± 15.0	57.1–71.2	64.7 ± 16.3	57.1–72.4	62.3 ± 13.2	56.1–68.4	58.1 ± 13.5	51.7–64.4
E post [pg∙mL^−1^]	62.3 ± 15.6	54.9–69.6	59.8 ± 14.4	53.0–66.5	62.6 ± 13.3	56.4–68.8	58.4 ± 13.3	52.2–64.6

Glu—glucose; [La^−^]—blood lactate; [H^+^]—hydrogen ions; pCO_2_—partial pressure of carbon dioxide; [HCO_3_^−^]—bicarbonate ions; BDNF—brain derived neurotrophic factor; T—testosterone; C—cortisol; E—epinephrine. *—statistically significant difference between pre-post (*p* ≤ 0.05).

## Data Availability

The data supporting the findings of this study are available from the corresponding author upon request.
